# Coriander (*Coriandrum sativum* L.) in Combination with Organic Amendments and Arbuscular Mycorrhizal Inoculation: An Efficient Option for the Phytomanagement of Trace Elements-Polluted Soils

**DOI:** 10.3390/microorganisms10112287

**Published:** 2022-11-17

**Authors:** Joël Fontaine, Jérome Duclercq, Natacha Facon, Dorothée Dewaele, Frédéric Laruelle, Benoit Tisserant, Anissa Lounès-Hadj Sahraoui

**Affiliations:** 1 Unité de Chimie Environnementale et Interactions sur le Vivant (UCEIV), Université du Littoral Côte d’Opale, UR 4492, SFR Condorcet FR CNRS 3417, 50 rue Ferdinand Buisson, CEDEX, 62228 Calais, France; 2 Unité Ecologie et Dynamique des Systèmes Anthropisés (EDYSAN, UMR7058 CNRS), Université de Picardie Jules Verne, 33 Rue St Leu, CEDEX, 80029 Amiens, France; 3 Centre Commun de Mesures, Université du Littoral Côte d’Opale, 59140 Dunkerque, France

**Keywords:** aromatic plant, compost, sewage sludge, aided phytostabilization, trace elements, soil microbial functional diversity

## Abstract

The cultivation of coriander (*Coriandrum sativum* L.) destined for essential oils production was recently presented as an innovative and economically viable alternative for the phytomanagement of trace elements (TE)-polluted soils. However, Cd accumulation in shoots has proven to be an obstacle in the valorization of the distillation residues and the development of these phytotechnologies. The present study aimed to evaluate the effect of arbuscular mycorrhizal fungus (*Funneliformis mosseae*) inoculation and organic amendment application on the soil TE bioavailability and plant uptake, as well as on the soil quality and health improvement. The application of compost and sewage sludge improved the growth of coriander and Cd and Zn immobilization in soil, resulting in reduced Cd plant uptake. A synergistic effect of arbuscular mycorrhizal fungi (AMF) inoculation and organic amendments was observed in the decrease in the extractable soil Cd and Zn concentrations, but not in the Cd plant uptake. Despite a significant decrease in Cd accumulation in shoots, coriander retained its accumulative phenotype, with a metal bioconcentration factor close to 1. Furthermore, both the vegetation and the organic amendments improved the soil quality and health by increasing its microbial biomass, as estimated by phospholipid fatty acids, soil enzyme activities (dehydrogenase, phosphatase, β-glucosidase, and cellubiosidase), and the bacterial metabolic function and diversity. The findings demonstrate the potential of *C. sativum*, particularly in combination with organic amendments and AMF inoculation, for the phytomanagement of TE-polluted soils and soil quality and health improvement.

## 1. Introduction

Soil health is critical for the appropriate functioning of terrestrial ecosystems. Unfortunately, anthropogenic activity-induced soil pollution adversely affects soil health at a global scale, with deleterious impacts on essential ecosystem services [1]. Typically, trace elements (TEs) pollution is a problem of great magnitude owing to their toxicity and persistence in soil, and their ability to negatively affect soil quality, plant growth, food quality, and animal and human health [2]. Therefore, the management of TE-polluted soils to reduce the associated risks has become a global priority [3]. Among the available soil-remediation techniques, conventional methods, such as soil washing or thermal stabilization, rapidly reduce the environmental risks associated with excessive TE concentrations, but are expensive and lead to the irreversible loss of soil and its beneficial ecosystem services [4]. Therefore, biological methods of soil remediation are now receiving more and more attention due to their ecological character, lower cost, and the gains in terms of aesthetic value [5]. Among green technologies, phytostabilization based on the use of TE-tolerant plants able to reduce the TE mobility could be an alternative management option to attenuate the environmental risks associated with TE-polluted soil and restore the soil fertility and ecosystem functions [6,7]. Furthermore, due to the difficult conditions in polluted sites related to the toxicity and mobility of TE, lack of required nutrients, poor water retention, or extreme pH, it is very difficult for some plant species to grow and/or immobilize TE. Hence, some amendments are needed to aid phytostabilization and improve the living environment of plants. Aided phytostabilization with organic amendments, such as sewage sludge, manure, or compost, can improve the soil fertility and, consequently, the amount of harvestable biomass, improve the physicochemical properties of soils, and potentially limit TE mobility and bioavailability in polluted soils [6]. Indeed, organic matter (OM) application allows TE availability reduction by increasing the soil pH and cation exchange capacity (CEC), and/or through the sorption and complexation of the metal fractions with OM [8]. Organic amendments also constitute an important source of carbon and energy for soil microorganisms and, therefore, stimulate their activities.

Some microorganisms can also facilitate the re-vegetation of TE-polluted soils. For example, arbuscular mycorrhizal fungi (AMF) are widely used in phytostabilization processes due to the benefits they provide to the plant, especially under conditions of water limitation, low fertility, and high levels of toxic elements [9]. Indeed, it has been described that AMF can decrease the TE concentration in plants by favoring metal immobilization through different mechanisms, such as the sequestration of TE by glomalin, a metal-sorbing glycoprotein, and TE immobilization in the wall of fungal hyphae and spores or storage in vacuoles [10,11]. Organic amendments can also facilitate AMF–plant symbiosis and improve plant growth, even under extreme conditions, such as those in TE-polluted soils [12,13,14]. Although AMF are obligate biotrophs and unable to use this carbon source, the positive effect of OM on the growth of the external mycelium has already been described [15,16]. OM introduction into the soil promotes plant growth and, therefore, carbon allocation to AMF, or can be also mediated through soil bacteria, which was reported to either directly enhance the germination and growth of extraradical AMF hyphae, or by indirectly influencing plant physiology [17].

The combination of AMF inoculation and organic amendments, as a technology for the phytomanagement of TE-polluted soils, has been little investigated, but there are indications that it is effective in ensuring the success of this management method [18]. For example, in a greenhouse pot experiment, Pérez et al. (2021) recently showed the interactive effect of compost application and AMF inoculation with *Claroideoglomus claroideum* on the growth of the metallophyte species *Oenothera picensis* and a decrease in Cu bioavailability in mine tailings soil [19]. In the same way, combined effects of AMF and composted pig manure on the growth of ryegrass and Cd and Zn uptake in soil polluted by electrical and electronic waste products have been shown [20]. The effectiveness of the combination of organic–biological amendments is generally demonstrated by a significant soil metal bioavailability reduction. However, their contribution to soil functionality restoration is generally not widely considered. It is, however, well established that the composition and functionality of soil microorganism communities appear to be an important ecological attribute that may indicate successful remediation of such TE-polluted soils [21].

The selection of appropriate plant species is another critical aspect of successful phytomanagement [22]. In particular, plants have to be able to grow in TE-polluted conditions and to develop vegetation cover in a relatively short period of time while producing high amounts of valuable biomass [23]. In recent years, the cultivation of aromatic plants destined for essential oil (EO) production has been presented as an innovative and economically viable alternative for reclaiming TE-polluted areas [24,25,26]. Essential oils are high-added-value products, widely used as aromatic agents in various non-food industries, such as perfumery, cosmetics, and medicine, as well as potential crop-protection products, which could provide economic benefits with the produced biomass [27]. Recently, two studies demonstrated the potential of coriander to be used for the phytomanagement of TE-polluted areas, given its tolerance to high soil TE concentrations and the presence of only trace amounts of TEs in coriander [26,28]. In these works, the valorization of distillation residues was envisaged in different ways for adding value chains, such as soil improvement, animal fodder, or anaerobic digestion. Unfortunately, coriander is a Cd accumulator, which limits the valorization of the distillation residues [28]. Indeed, Cd is recognized as one of the most hazardous pollutants due to its severe toxicity and high mobility in soil. This carcinogenic metal is, therefore, readily available for plant uptake and can easily integrate into the food chain. Therefore, it is of great importance to limit its bioavailability in the soil [29]. In this context, we hypothesized that a combined organic–biological amendment into TE-polluted soils would improve plant growth, reduce the Cd accumulation in coriander shoots, and restore the soil functions by increasing the soil microbial biomass and activities, as well as their functional diversity. To validate this hypothesis, we evaluated, in a pot experiment, the combined effect of organic amendments (compost and sewage sludge) and biological amendment with the AMF *Funneliformis mosseae* on the phytostabilization of aged TE-polluted soil using an aromatic plant, *Coriandrum sativum* L. The effects of these amendments on plant growth, the bioavailability of TEs in soil, TE accumulation in the plant, and on the soil microbial biomass (quantified via specific lipid marker measurements) and functionality (community-level physiological profiles and soil enzyme activities) were measured to evaluate the sustainability of this practice in recovering the functioning of this TE-polluted soil.

## 2. Materials and Methods

### 2.1. Soil Description and Sampling

The soil samples used in this experiment were collected at Evin-Malmaison in northern France (50°25′55.5″ N, 3°02′25.5″ E) from a former agricultural field, 600 m north and under the wind of the former foundry Metaleurop Nord. This smelter was one of the major Pb plants in Europe for around 100 years, producing significant amounts of lead-contaminated dust, which deposited on the surrounding soils and led to significant contamination [30]. In December 2019, all soil samples were obtained by mixing subsamples collected from different zones of the field area at a depth of 0–20 cm. Subsequently, the soil was homogenized, air-dried at room temperature, and finally passed through a 4 mm mesh sieve. The soil texture consisted of silty loam, with a slightly alkaline water pH (7.9) (Table 1). The topsoil was characterized by high total concentrations of Cd, Pb, and Zn (5, 326, and 409 mg/Kg, respectively). These concentrations are approximately 12, 10, and 6-fold higher, respectively, than those reported in the regional background levels for agricultural soils [31].

### 2.2. Amendment Addition and Pot Experimentation

Two organic amendments and one biological amendment (AMF inoculum) were tested in this study. The sewage sludge used was collected from Calais (France) treatment plant and composted in an automated fast-composting system. The compost was obtained from the dehydration of the digestate (liquid and solid residues) after the anaerobic fermentation of plants and OM by methanization. The composting process lasted for 2 months, during which the open-air heaps were turned regularly. Based on phytotoxicity bioassays for maturity assessment [32], both of these organic amendments can be considered as mature and non-phytotoxic products, as their germination indexes (GIs) were higher than 50% (Table 1). Briefly, to determine the GI, lettuce (*Lactuca sativa* L., ‘Appia′) seeds were germinated in Petri dishes containing 5 mL of 20% (*w*/*v*) water-soluble extracts of compost under darkness at room temperature for 72 h. The phytotoxicity was evaluated by monitoring the emergence of seedlings (20 seeds), the number of germinated seeds at 24 h, and growth of roots at 72 h using the following equation: GI% = (NGE × LRE)/(NGW × LRW) × 100. NGE and NGW correspond to the number of seeds germinated in water-soluble extracts and distilled water, respectively. LRE and LRW are the rootlets’ lengths in soluble extracts and distilled water, respectively.

The mycorrhizal inoculum (*Funneliformis mosseae*) was purchased from MycAgro (Bretenière, France), which is a mix of spores, mycelium and mycorrhizal root pieces and contains as a minimum 10 propagules/g. The physico-chimical parameters that characterize the soil and the organic amendments were analyzed by Cesar laboratory (Ceyzeriat, France) according to the method of the French standard AFNOR (granulometry (NFXNFX 31-107), pH (NFX 31-117), Total organic carbon (ISO 10694), azote (ISO 13878), CEC (NFX 31-130), micro and macro elements (NFX 31-108, NFX 31-160, NFX 31-120 and NFX 31-122)) and are shown in Table 1. The organic amendments were rich in mineral nutrients (Available P Olsen: 0.9 and 2.9 g/Kg; total N: 15.1 and 40.9 g/Kg for compost and sewage sludges, respectively) and OM (355 and 442 g/Kg for compost and sewage sludges, respectively) with an alkaline pH (7.11 and 8.92, respectively). TE concentrations in both compost and sewage sludges were below the limits established for agricultural use of compost (Cd < 3, Cr < 120, Cu < 300, Ni < 60, Pb < 180 and Zn < 600 mg/Kg; NF U 44-051) and sewage sludges in France (Cd < 20, Cr < 1000, Cu < 100, Ni < 200, Pb < 800 and Zn < 300 mg/Kg; NOR ATEE9760538A). The soil was then mixed with 4% (w/w) of the sewage sludge or compost. A non-amended soil was used as the control soil. The amounts of the introduced amendments were calculated on the dry weight of the soil. After three weeks of preliminary chemical stabilization of amended soils, 18 microcosms (1 L, diameter 10 cm), 6 per condition (non-amended, amended with compost and with sewage sludges), were filled with 720 g of soil and mixed with 80 g (10%) of the commercial AMF inoculum inoculated condition (I) and 18 others microcosms (6 per condition) were filled with 720 g of soil and mixed with the equivalent mass of autoclaved AMF inoculum (non-inoculated condition, NI). Next, half of these microcosms were vegetated or not (non-vegetated) with 10 seedlings of coriander (*Coriandrum sativum* L.). In total, the experiment was set up with 3 modalities (non-amended, amended with compost or with sewage sludges) × 2 AMF inoculation (NI, I) × 2 plant conditions (non-vegetated, vegetated) for 3 months of culture in the controlled conditions at 20 °C with supplemental light (15,000 Lux, 12 h/12 h). The pots were watered with deionized water every two days to keep soil moisture close to 60% of field capacity and randomized every seven days.

### 2.3. Sampling and Measurements

#### 2.3.1. Plant Analysis

After 3 months of culturing, the roots and shoots (stem and leaves) of 30 plants were collected. The shoots were lyophilized within 48 h and weighed as biological replicates to determine the dry weights. The roots were rinsed with sterile deionized water, cleared in KOH (10%, *w*/*v*) for 10 min at 90 °C, and stained with trypan blue (0.05%, *w*/*v*) as described by Phillips and Hayman [33] and modified by Koske and Gemma [34]. The stained root fragments were stored at 4 °C in a water/glycerol/lactic acid (1:1:1, *v*/*v*/*v*) solution until observation. The percentage of AMF colonization was determined by the microscopic observation of stained root samples using the method of McGonigle [35]. One hundred and thirty-five root fragments of 1 cm from each different pot were observed under a microscope (×100 magnification, Nikon Eclipse E600, Nikon, Tokyo, Japan). Three grid-line intersections per root fragment were examined. In total, more than 400 observations per treatment were analyzed.

#### 2.3.2. pH Measurement

The soil pH was measured following the NF ISO 10390 (2005) standard. Five grams of 2 mm-sieved soils were mixed with 25 mL of distilled water and shaken for 2 h. The water pH was measured after 1 h of resting.

#### 2.3.3. TE Analysis

For total TE determination, soil samples were microwave-digested (Multiwave 3000, Anton Paar, St Albans, UK) following NF-EN-13656. Briefly, 0.5 g of soil was digested at 180 °C for 20 min in 2 mL of 67% nitric acid, 6 mL of 36% hydrochloric acid, and 2 mL of 48% hydrofluoric acid. The extract was then filtered using 0.45 mm Whatman^®^ filter paper and stored for analysis. To estimate the easily soluble fraction (water-soluble compounds, exchangeable bound ions, and readily soluble metal complexes) of TEs in soil, selective extraction was performed with 1M NH_4_NO_3_ following the NF ISO 19730:2008(E). After 2 h of shaking, the soil extract mixtures were filtered through a 0.45 mm nylon filter and the leachates were acidified to pH 2 and stored for analysis. Traces elements were analyzed in all plant samples (0.2 g dry wt) after digestion at 180 °C for 20 min in 10 mL of 67% nitric acid and 3 mL of ultra-pure water using a microwave digester (Multiwave 3000, Anton Paar, St Albans, UK)). The total As, Cr, Cd, Cu, Ni, Pb, and Zn concentrations were measured using an inductively coupled plasma–optical emission spectrometer (ICP-OES, ICAP 6300 DUO, ThermoFisher Scientific, Loughborough, UK) and an inductively coupled plasma–mass spectrometer (ICP-MS, Varian 820 MS, Varian, Palo Alto, CA, USA), depending on the sample concentrations. Two standard reference materials were included for analytical quality control (BCR-679, white cabbage, and ERM-CC141, loam soil, Sigma Aldrich, St Louis, MO, USA). The recovery rates ranged from 100–106% for Zn, 103–112 for Cu, 87–91% for Cd, and 83–92% for Pb. For the other elements, the recovery rates ranged from 87% to 112%.

#### 2.3.4. Phospholipid Fatty Acid (PLFA) Analysis

The PLFAs were extracted from 3 g of freeze-dried soil samples with a mixture of chloroform–methanol–phosphate buffer (1:2:0.8, *v*/*v*/*v*) as previously described by Frostegård et al. [36]. The lipids were fractionated in solid-phase extraction columns containing silica (6 mL, 500 mg sorbent, Interchim, France) into neutral lipids, glycolipids, and polar lipids containing phospholipids by successive elutions of chloroform, acetone, and methanol (1:2:1, *v*/*v*/*v*) [36]. The PLFAs were then trans-esterified using 0.2 M KOH in methanol. The resulting fatty acid methyl esters were analyzed with a gas-phase chromatograph–mass spectrometer (QP-2010 Ultra, Shimadzu, Kyoto, Japan) equipped with a single quadrupole mass detector simultaneously coupled with a flame ionization detector (FID). Samples were analyzed in a split mode (ratio 80:1) on a ZB-1MS fast capillary column (100% dimethylpolysiloxane, 10 m length × 0.1 mm inner diameter × 0.1 µm phase thickness, Zebron, Phenomenex, Torrance Calif, CA, USA) using helium as a carrier gas at a constant linear velocity (40 cm/s). The injector temperature was 280 °C, while the detector temperatures were 330 °C for FID and 280 °C for the ion source. The initial temperature was 175 °C, which increased by 25 °C/min to a final temperature of 275 °C for 0.5 min. The ionization mode was electronic impact at 70 eV, and the mass range between 50 and 400 *m*/*z* was scanned. The single-impact monitoring mode was used simultaneously.

Fatty acid methyl ester quantification was performed using nonadecanoic acid methyl ester as an internal standard (Sigma Aldrich). The fatty acids were identified by comparing their relative retention times to those of commercial standards (C4-C24:1, Sigma Aldrich) and comparison with spectra that were either obtained from a commercial standard and/or reported in the literature (NIST Standard Reference Database).

The soil microbial biomass was determined using specific PLFAs. Saprotrophic fungal biomass was estimated based on the 18:2ω6,9 content [37]. The sums of i15:0, a15:0, i16:0, i17:0, a17:0, and cy17:0, and C18:1ω7 and cy19:0 were quantified as indicators of Gram-positive and negative bacterial biomasses, respectively [36]. AMF biomass was estimated with the PLFA 16:1ω5 content [38]. The sum of the PLFA content was used to estimate the total microbial biomass and the sum of cyclopropyl PLFA to the sum of their monoenoic precursors (cy17:0 + cy19:0)/(16:1ω7 + 18:1ω7) was used as an indicator of physiological or nutritional stress in bacterial communities [39].

#### 2.3.5. Community-Level Physiological Profiles

The metabolic potential of soil communities was performed according to the community-level physiological profiles (CLPP) using Biolog EcoPlates^TM^ (Biolog Inc., Hayward, CA, USA) [40]. For each soil sample, an EcoPlate containing 31 different carbon sources (and a blank with no C source) in triplicate was inoculated and incubated at 25 °C for 196 h in an OmniLog^®^ System (Biolog Inc.). The rate of carbon source utilization was indicated by the reduction of tetrazolium, a redox indicator dye, which changes from colorless to purple and is detected at a wavelength of 590 nm. Data were recorded every 15 min of incubation and saved in OmniLog units, generated by the Biolog Data Analysis software (v1.7, Biolog Inc). The values for each well were calculated by subtracting the blank well values from each plate well. Absorbance at a single time point of 70 h was used for comparisons, following the recommendation of Weber et al. [41]. The metabolic potential of the soil microbial communities of each sample, expressed as average well color development (AWCD), was calculated at the determined time point by dividing the sum of the optical density data by 31 (number of substrates). The total number of wells in a replicate with an absorbance above 25 OmniLog units was counted to define the functional richness of the soil microbial community.

#### 2.3.6. Soil Enzyme Activities

We used 4-methylumbelliferyl (MUB)-β-D-glucopyranoside (BG), 4-MUB-β-D-cellobioside (CB), 4-MUB-phosphate (PHOS), and 4-MU-sulfate (SUL) to determine the β-glucosidase, cellubiosidase, phosphomonoesterase, and arylsulfatase activities, respectively. These enzymatic activities were determined according to the fluorescence-based enzyme protocols [42], which measured the fluorescence of reaction products MUB (4-methylumbelliferyl). We added 1.5 g of fresh soil to 50 mL of 50 mM sodium acetate (pH = 7.65), and the whole mixture was homogenized for 15 min. Then, 200 μL of soil slurry was added to a 96-well microplate with 50 µL of appropriate 200 μM substrate. After 3 h of incubation at 25 °C, we measured the enzyme activities with a microplate reader (Infinite M1000 PRO, Tecan System) using an excitation wavelength of 365 nm and an emission wavelength of 450 nm, and compared them with a standard dilution curve of MUB (0 to 100 µM).

Soil dehydrogenase activity was measured as described in [43]. Four grams of fresh soil were mixed with CaCO_3_ to a final mass ratio of 100:1 and incubated for 24 h at 37 °C with a solution of 1.33% (*w*/*v*) 2,3,5-triphenyl tetrazolium chloride. The resulting triphenylformazan was then extracted with methanol, measured at 485 nm (Eon spectrophotometer, BioTek Instruments Inc., Winooski, VT, USA), and compared with a standard dilution curve of triphenylformazan (0 to 400 µg).

### 2.4. Statistical Analysis

Statistical analyses were performed using XLSTAT 2021.1.1 software (Addinsoft 2021 Paris, France). Data expressed in percentages, such as the AMF colonization rates, were converted to arcsine values before statistical analysis (ASIN function on Microsoft^®^ Excel 16.50). The Shapiro–Wilk and Breusch–Pagan tests were used before any statistical analyses to verify the normality and the homoscedasticity of the data, respectively. According to the results of the previously mentioned tests, non-parametric statistical tests (Kruskal–Wallis) were chosen for the comparison of the biomass data, mycorrhization rates, CLPP, and soil enzyme activities. To analyze the effect of organic amendments, AMF inoculation, and vegetation on other parameters, comparisons of means were performed using one-way analysis of variance (ANOVA) and two-way ANOVA using the Type III sum of squares. The comparison of means was performed using the Tukey test at three levels of significance: * *p* < 0.05, ** *p* < 0.01, and *** *p* < 0.001. These data were also subjected to principal component analysis (PCA) to determine whether the soil parameters were different according to the amendment or vegetation. This analysis was performed with R (v4.0.4; http://www.r-project.org/ accessed date 14 November 2022) using the standardized data (scale function) and the *dudi.pca* function of the *vegan* R package [44]. We created the PCA biplot using the *fviz_pca_biplot* function from the *factoextra* R package [45]. To test the significance of the biological parameters and soil TE contents, we ran permutational multivariate analyses of variance (perMANOVA) using the *adonis* function from the *vegan* R package.

## 3. Results

### 3.1. Effects of Organic Amendments and AMF Inoculation on Soil Chemical Parameters and Plant Growth

While the application of both organic amendments to the initial soil samples resulted in a significant increase in the available P Olsen and Mg contents, an increase in the total organic carbon and OM contents was only observed upon compost application (Table 1). The introduction of these organic amendments to the soil did not change the soil structure or pH. Low soil acidification was observed after 3 months of cultivation under all conditions, except for the soil amended with sewage sludge, where the pH values were lower than 7.1 for the non-vegetated soils (inoculated and non-inoculated) and 7.3 for the vegetated and non-inoculated soils (Figure S1). Under non-inoculated conditions, compost and sewage sludge improved the average plant dry weight by 120 and 100%, respectively (Table 2). We observed a weaker increase (38%) when the compost was paired with the AMF and no improvement if the double amendment included sewage sludge and AMF. Compared with the non-inoculated and organic amendment conditions, AMF inoculation did not promote plant growth nor root colonization by *F. mosseae*, which ranged between 49 and 56%.

### 3.2. Effect of Organic Amendments and AMF Inoculation on TE Concentration in Soil and Plant Metal Uptake

**Figure 1:**
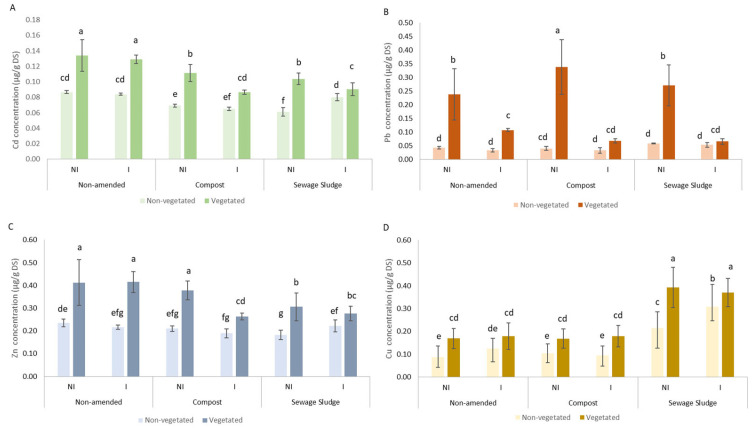
NH_4_NO_3_-extractable concentrations (µg/g dry soil (DS)) of Cd (**A**), Pb (**B**), Zn (**C**), and Cu (**D**) in soil amended or not (unamended) with compost or sewage sludge in the presence (I) or not (NI) of arbuscular mycorrhizal inoculum. Significant differences between conditions are indicated by different letters at the level of α = 0.05.

The total concentrations of TE (As, Cr, Cd, Cu, Ni, Pb, and Zn) were measured in the soil and shoots of coriander (Table S1) and Table S2. Interestingly, the application of 4% organic amendment only resulted in a significant decrease in the Pb content in non-inoculated soil (Table S1). The soil vegetation did not modify the total TE concentration in the soil (Table S1). However, the vegetation increased the extractability of Cd, Zn, and Pb in soil without amendment (whether organic or biological) and that of Cu in non-inoculated soil from 13 to 60% (Figure 1). The AMF inoculation decreased the extractability of Pb under all vegetated conditions and that of Cd and Zn only in organically amended vegetated soil. In non-vegetated soil, AMF inoculation increased the extractable concentrations of Cd, Zn, and Cu only in soil amended with sewage sludge. We observed, in soil with compost application, lower extractability of Cd, Zn (except in the non-inoculated and non-vegetated condition), and Cu. This amendment induced an increase in the extractable Pb under non-inoculated and vegetated conditions. The application of sewage sludge caused a decrease in the extractable soil Cd and Zn (except under the non-vegetated and inoculated conditions). In contrast, the organic amendment induced an increase in the extractable Cu and Pb under the non-vegetated and non-inoculated conditions.

**Figure 2:**
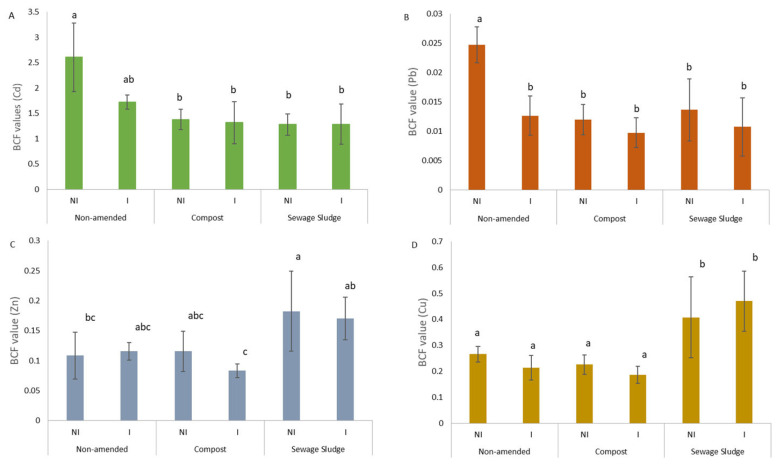
BCF values calculated as the plant part metal concentration over the total metal concentrations: Cd (**A**), Pb (**B**), Zn (**C**), and Cu (**D**). Significant differences between conditions are indicated by the different letters of α = 0.05.

The predominant TEs detected in coriander shoots under the non-amended and non-inoculated conditions were Cr < Ni < Cu < Pb < Zn < Cd (Table S2). Regardless of the TE considered and the conditions (amended and non-amended), AMF inoculation did not induce a change in the TE shoot contents. The metal bioconcentration factor (BCF) calculated with the mean TE concentrations in shoots and soil (Figure 2) indicated an accumulator response of coriander for Cd (BCF = 2.61) only under the non-amended and non-inoculated condition, and an excluder response for Pb (BCF = 0.02), Zn (BCF = 0.11), and Cu (BCF = 0.27). A decrease in the BCF value was observed for Pb under the amended conditions and under the non-amended and inoculated conditions. The presence of sewage sludge in soil induced an increase in the Zn and Cu BCF values.

### 3.3. Effect of Organic Amendments and AMF Inoculation on the Soil Microbial Biomass

Soil vegetation significantly increased the biomass of all microbial groups (Table 3), including the Gram-positive bacteria (*p* value < 0.01), Gram-negative bacteria (*p* value < 0.001), saprotrophic fungi (*p* value < 0.001), and AMF (*p* value < 0.001). The application of organic amendments to the TE-polluted soil induced an increase (*p* value < 0.01) in the bacterial biomass (Gram-positive and -negative) and total microbial biomass (*p* value < 0.001). AMF inoculation did not influence the soil microbial biomass. The combined treatment involving vegetation and organic amendments resulted in significant differences in the AMF biomass (*p* value < 0.05). In order to evaluate the impact of the interaction of vegetation, organic amendment applications, and AMF inoculation during the experiment on the physiological status of the soil microbial communities, different ratios, such as the fungal/bacterial, fungal/AMF, and Gram-positive/Gram-negative bacterial biomasses, were calculated, but no significant difference was observed for these parameters. Only a significantly lower value of the stress indicator (*p* value < 0.001) in the vegetated soil was observed.

### 3.4. Effect of Organic Amendments and AMF Inoculation on the Soil Microbial Community Functionality

The lowest AWCD level, used as a proxy of the metabolic potential of soil microbial communities, was detected in the non-amended and non-vegetated soils (Table 4). This profile was mainly based on metabolized sources, such as carbohydrates, carboxylic acids, and amino acids. Only the application of sewage sludge stimulated this metabolic potential. However, the presence of coriander strongly stimulated this activity in non-amended soil and soil amended with compost. The same profile was observed for the soil functional richness, with an increase in this index when the soil was vegetated (regardless of the conditions) and upon sludge amendment. A similar profile was found for the dehydrogenase activity. However, in non-amended soils, the presence of coriander did not induce an improvement in the dehydrogenase activity. This increase was observed only when the soil was amended and vegetated. Organic amendments influenced the β-glucosidase activity, with the highest value of this activity detected in vegetated soils amended with sewage sludge. The phosphomonoesterase activity was not affected by the amendments, nor by the soil vegetation. The highest values were observed in non-amended and vegetated soils. Concerning β-cellobiosidase and arylsulfatase, their activities were not affected by the application of organic amendments, nor by the soil vegetation. The highest values were observed in non-vegetated soil and soil amended with sewage sludge and AMF inoculum. For the activities of all these enzymes, no significant difference was observed between the inoculated and non-inoculated conditions.

### 3.5. Relationships between Biological Parameters and Soil TE Contents

**Figure 3:**
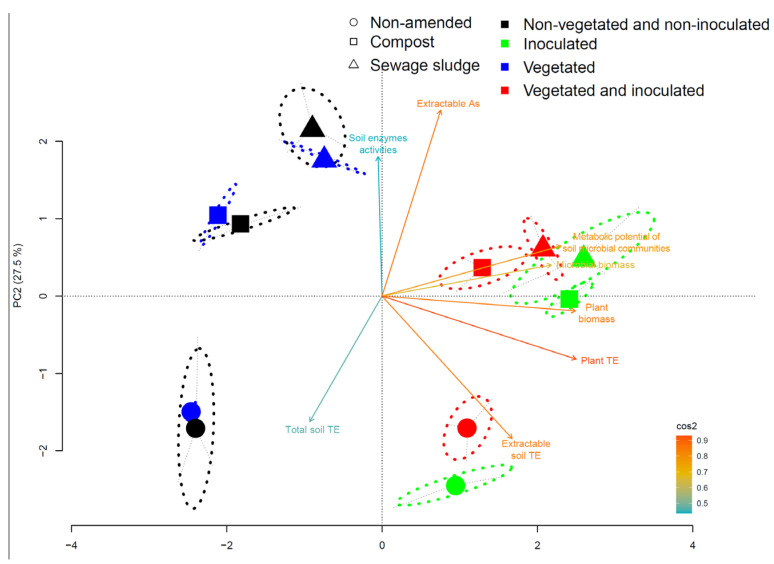
Principal component ordination of all of these parameters (microbial biomass, plant biomass, TE extractable concentration in soil, total TE concentration in soil, TE concentration in shoots, and soil enzymes’ activities) in the different modalities (non-amended or amended with compost or with sewage sludge, AMF inoculation (NI or I), and cultivation conditions (non-vegetated or vegetated).

The principal component ordination of all of these parameters (Figure 3) indicated a distribution of the different conditions along two axes covering 72.2% of the variance. Axis 1 (50.8%) was characterized by the metabolic capacities of the soil microbial communities and the microbial and plant biomass. This axis was also positively correlated with the TE levels in plants. Axis 2 (21.4%) was characterized by two sets of parameters. The first set was composed of the soil enzymatic activities, while the second set included the soil TE contents and their extractability. These two sets were negatively correlated along axis 2. Non-amended soils were associated with axis 2 and with the soil TE contents. Organic amendments influenced the soil TE levels (perMANOVA, F = 3.43, *p* value = 0.03) and induced a shift in the soil parameters along axis 2. These amendments also affected the soil enzyme activities (perMANOVA, F = 13.849, *p* value < 0.01) and strengthened the link between this parameter and the soils that only received organic amendment. Biological amendment influenced the soils along axis 1, mainly by promoting the microbial communities (perMANOVA, F = 1784.28, *p* value < 0.01) and the extractability of TE (perMANOVA, F = 42.477, *p* value < 0.01). This input also influenced the soils along axis 2 by negatively modulating the soil enzymes’ activities (perMANOVA, F = 27.761, *p* value < 0.01). When the organic amendment was associated with a biological input, the soils evolved similarly to that observed following the organic amendment, also with an influence on the soil enzymes’ activities (perMANOVA, F = 15.105, *p* value < 0.01). The vegetated soil by coriander induced a logical change concerning the plants’ TE uptakes. These treatments influenced other parameters, such as the microbial communities (perMANOVA, F = 17.32, *p* value < 0.01), their activities (perMANOVA, F = 20.737, *p* value < 0.01), and the soil enzymes’ activities (perMANOVA, F = 87.768, *p* value < 0.01), as well as the TE extractability (perMANOVA, F = 62.904, *p* value < 0.01). These changes in the different parameters analyzed positioned the vegetated soils in the ordination very close to the unamended soils. The vegetated and organically amended soils progressed similarly to that observed upon organic amendment. The association between biological amendment and vegetation induced a change along axis 1 with an influence on the TE levels in the soil (perMANOVA, F = 3.436, *p* value = 0.02), the TE extractability (perMANOVA, F = 28.037, *p* value < 0.01), and the soil enzymes’ activities (perMANOVA, F = 31.235, *p* value < 0.01). The combination of these three inputs induced a change combining the different responses and shifted the soils along both axis 1 and axis 2 of the ordination.

## 4. Discussion

After 3 months of cultivation, the TE concentrations in coriander shoots, as well as the total and extractable TE concentrations in rhizosphere soil, were quantified. With values between 5.87 and 12.44 mg/Kg and a BCF of >1, Cd was the predominantly accumulated element in the coriander shoots. These concentrations measured were above reported concentrations in non-polluted [46,47] as well as polluted [28,48] soils and the limit (i.e., 0.1 mg/Kg) in vegetables recommended by FAO/WHO (2011) or the EU regulation 2021/1323. These findings confirm the Cd accumulator phenotype previously observed for this aromatic plant species [28]. Our results also showed an excluder response for Zn, Pb, and Cu with a slight increase in the Zn and Cu BCF values when plants were cultivated in the presence of sewage sludge. This greater accumulation in shoots was probably related to the high concentrations of these two elements in this organic amendment, although they were below the limits established for agricultural use. TE accumulation in plant tissues has frequently been reported in plants grown on sludge-amended soil. For example, higher amounts of Fe, Cu, and Zn were absorbed in maize and barley grown on sludge-amended soil than in those grown on unamended soil [49].

The application of both organic amendments induced an overall decrease in the bioavailability of Cd and Zn in soil (extractable with 1 M NH_4_NO_3_) and in the accumulation of Pb and Cd in coriander shoots. However, despite the nearly 50% decrease in the Cd BCF values in the presence of organic amendments, the Cd accumulator phenotype of coriander was, regrettably, maintained. The introduction of organic amendments into soil has been shown to be efficient in the restoration of polluted soil, as it can improve the immobilization of metal(loids). Several studies have shown that compost [50,51,52] or sewage sludge [53,54] can reduce the mobility, bioavailability, and plant uptake of these TEs. This immobilization is related to various complex processes (e.g., adsorption onto mineral surfaces), the formation of stable compounds with organic ligands, surface precipitation, and ion exchange [55]. However, these processes are still not well understood and their effectiveness may vary depending on the soil properties, the type of organic amendments, and application practices [56]. In a recent review, Palansooriya et al. [55] described, in detail, the contribution of these amendments to TE immobilization. The authors concluded that compost has varying effects on TE immobilization, depending on the material maturity levels and composition, as well as the soil properties. They recommended applying mature compost to minimize the TE mobility effect caused by increased dissolved organic matter (DOM). Additionally, the formation of dissolved organo-metallic complexes with sewage sludge-derived DOM can lead to a decrease in TE sorption in soil. Therefore, the sewage sludge-derived DOM content and soil properties should be considered before application to soil. In our study, although the application of both organic amendments to the initial soil samples did not induce changes in the soil structure and pH, the total and organic C, P, and Mg contents increased with the application of compost, which can not only improve TE immobilization in soil, but also the soil nutritional state for plants [57]. Indeed, we also showed that the application of organic amendments improved coriander shoot growth overall. This improvement is in accordance with previous studies showing that the application of these organic amendments enhanced plant growth in TE-polluted soil [58,59,60]. It is well known that the application of organic amendments generates a better soil nutritional status [61]. Phosphorus is the second-most-important nutrient after nitrogen in plant development. Additionally, its increase in amended soil could be closely linked to the observed plant biomass’s improvement [58]. The introduction of organic amendments also changed the soil environment, which is also in favor of plant growth [62]. It is also possible that the improvement in plant growth is due to the observed TE immobilization in soil and, consequently, a reduction in their toxicity [63].

The soil vegetation with coriander caused an overall increase in the extractable soil Cd, Pb, Zn, and Cu concentrations. Some studies showed that plant roots, aided by plant-produced chelating agents and plant-induced pH changes and redox reactions, are able to solubilize and take up micronutrients and particularly ions at very low levels in the soil. In this experiment, we showed that vegetation did not cause a significant change in the pH or CEC. On the other hand, it is likely that, to manage nutrient bioavailability and cope with environmental metal stresses, aromatic plants, such as coriander, release numerous metabolites into the rhizosphere [64]. In a recent review, Ait Elallem et al. [24] described the capacity of aromatic plants to change the physicochemical properties of the rhizosphere soil, as well as the availability of TEs in soil. In 2021, Saleem et al. [65] showed an increase in the organic acid contents (i.e., oxalic, malic, formic, citric, acetic, and fumaric acid) in coriander roots exposed to an increasing level of boron in the soil. These low-molecular-weight organic acids were probably the most important exudates that influenced the TE availability in soil by either forming complexes with metal ions or by modifying soil characteristics. The TEs that are available for plant uptake are those that are present as soluble components in the soil solution or those that are easily solubilized by root exudates.

In contrast, the plant AMF inoculation did not significantly affect the shoot growth. Similarly, the combined organic–biological amendments did not produce an additive or synergistic effect on plant growth. However, in the presence of organic amendments, a more significant decrease in the extractable Cd and Zn was observed when the plants were inoculated with the AMF *F. mosseae* revealing a synergistic effect of these two amendments. We showed that this combined treatment resulted in a significant increase in AMF biomass. It has been described that AMF can increase TE immobilization in soil through different mechanisms, such as the sequestration of TE by glomalin or TE immobilization in the wall of fungal hyphae and spores or their storage in vacuoles [10,11]. It is well established that the plant growth response would depend on the identities of both the plant and the AMF species [66,67]. In our experiment, the mycorrhizal rates were overall the same under all conditions (I and NI plants). Therefore, it was difficult to assess the coriander-colonization capacities of the AMF used in our experiment, *F. mosseae*. In a greenhouse pot experiment, Fatemi et al. [68] showed, despite the low colonization rates, that the mycorrhizal symbiotic relationship between coriander and *F. mosseae* was possible under Pb stress conditions and allowed an improvement in plant growth at Pb concentrations above 1000 ppm. Coriander is able to establish mycorrhizal symbiosis with the native AMF present in the non-sterilized soil used in this experiment [26]. Unlike our results, some studies have also shown, despite the spontaneous mycorrhization, that the introduction of AMF commercial inoculum to TE-polluted soil could result in a significant improvement of the total mycorrhizal rates, indicating variable responses across experiments [26,69]. In contrast, a more significant decrease in the extractable Cd and Zn was observed in inoculated plants. AMF could improve TE immobilization through different mechanisms, such as production in the mycorrhizosphere of glomalin-related soil proteins, TE accumulation in fungal structures (vacuoles or fungal vesicles in mycorrhizal roots), and TE adsorption by extraradical hyphae [10]. Many studies have demonstrated a synergistic effect of AMF inoculation and organic amendments on TE immobilization in soil [19,70,71]. Some studies described shifts in the AMF community as a consequence of the application of different organics amendments [72,73]. The impact of fertilization regimes on the AMF community composition was correlated with the OM composition in rhizosphere soil [74]. Several studies have also demonstrated that plant-associated microorganisms can contribute to the reduction in TE uptake by plants [75,76]. In our experiment, the decrease in the bioavailability of Cd and Zn in soil and in the accumulation of Pb and Cd in coriander shoots could be attributed to a change in the soil microbial communities. Indeed, it has also been shown that rhizosphere microorganisms can regulate plant uptake and TE bioavailability in polluted soils by various processes of oxidation, reduction, complexation, immobilization, adsorption, and dissolution [77]. For example, Wang et al. [78] showed reduced Cd uptake by Oryza sativa and Cd bioavailability in soil inoculated with Cd-tolerant *Pseudomonas* TCd1. Even if the application of bacterial inoculants in combination with soil amendments in an assisted phytostabilization process has still not been extensively developed, Siebielec et al. [79] showed that the effectiveness of organic amendments in the phytostabilization of strongly polluted smelter deposits can be enhanced by plant-growth-promoting bacteria and that the interaction of the soil amendments and some bacterial strains stimulated a decrease in the TE extractability, likely through their phosphate-induced precipitation.

Coriander cultivation, AMF inoculation, and organic amendment application can also play a crucial role in restoring soil functionality that was disturbed by metal contamination. Indeed, microbial communities have an important role in many soil processes (e.g., OM formation and decomposition, respiration, and nutrient cycling) and the delivery of essential soil ecosystem services [80]. Owing to their sensitivity and their capacity to provide information integrating many environmental factors, the various biological parameters tested (microbial biomass, enzymatic activities, and CLPP) were proven to be good indicators of soil health and, therefore, quality [53,81]. Numerous studies have shown a reduction in the abundance, activity, and diversity of soil microbial communities after exposure to TE [53,82]. In our agricultural polluted soil samples, the relatively low fungi-to-bacteria ratios suggested an extensively managed soil related to tillage, high rates of fertilization, and a low C:N ratio, favoring bacteria [83,84,85]. In addition, the total microbial biomass in the soil samples, based on the sum of both bacterial- and fungal-specific PLFA markers, increased with soil vegetation and the application of organic amendments. Plants are able to secrete specific root exudates and rhizodeposits, and to shape the microbial communities’ structures in the rhizosphere [86]. Aromatic plant species, such as coriander, are also susceptible to releasing secondary metabolites with antimicrobial properties in soil; therefore, they could play an important role in the construction of bacterial communities [86,87]. In 2021, Choudhary et al. [88] studied the bacterial diversity and the biochemical properties in the rhizosphere soils of cumin and coriander. The authors showed, by metabarcoding approaches, that the microbial profile was directly correlated with crop root exudates and edaphic factors, such as soil organic carbon and nitrogen. In this study, the cyclopropyl-to-cyclopropyl precursor (cy17:0 + cy19:0/16:1ω7 + 18:1ω7) PLFA ratio was used as an indicator of physiological or nutritional stress for microbial communities. A higher precursor ratio is generally associated with a decrease in bacterial growth rates and an increase in carbon and nutrient limitation. The significantly lower values of this stress indicator in the vegetated soil suggested that vegetation contributed to the decrease in stress factors affecting the microbial communities. In the same way, organic carbon and other nutrients provided by organic amendments feed telluric microbes and can have significant effects on the soil microbial activity, biomass, and composition [89]. In a recent study, Rodríguez-Berbel et al. [90] showed that soil bacterial communities are clearly influenced by the types of organic amendments applied. The authors showed that soils amended with sewage sludge, with a higher content of labile OM, promoted the proliferation of specific copiotrophic bacterial taxa, such as *Aminobacter*, *Taibaiella,* or *Pseudomonas*. They explained that these taxa could play an essential role in the carbon and phosphorus cycles, and that the high activities of β-glucosidase, dehydrogenase, and phosphatase could contribute to the rapid consumption of soil nutrient reserves. In contrast, they showed that soils amended with compost and a hardly biodegradable OM were characterized by the highest microbial diversity and activity, guaranteeing a slower release of essential nutrients for soil fertility. In our experiment, a predominance of Gram-negative versus Gram-positive bacteria was observed under all conditions and organic amendments increased both bacterial groups, with a prevalence of Gram-negative bacteria in vegetated soil. It has been proposed that Gram-negative bacteria are able to adapt easier to adverse conditions, such as those of polluted soils [91,92], and use more plant-derived C sources that are relatively labile, while Gram-positive bacteria use C sources derived from soil OM that are more recalcitrant [93]. On the other hand, we also showed that ratios, such as fungal/bacterial, fungal/AMF, and Gram-positive/Gram-negative bacterial biomasses, did not vary between treatments. These results seem to indicate some stability of the microbial biomass, despite all of these treatments. In our experiment, similarly, hydrolytic soil enzymes, such as dehydrogenase phosphatase, β-glucosidase, and cellubiosidase, increased after the addition of both organic amendments, especially with sewage sludge. These hydrolytic enzymes enhanced the degradation and stabilization of various organic substrates in soil and could also influence the availability of essential nutrients to plants and microorganisms. Therefore, they could be used as essential indicators for evaluating the quality and health of polluted soil [94]. As an example, the β-glucosidase activity has been found to be sensitive to soil management and has been proposed as a soil quality indicator, because it provides an early indication of changes in the OM status and its turnover [95]. Regarding functional diversity, the AWCD and functional richness parameters were significantly higher in the organic-amended and vegetated soils. These results suggest that telluric microorganisms tended to have greater rates of utilization for all groups of carbon sources after the introduction of organic amendments. It is well established that the metabolic profile and functional diversity indexes of soil microbial communities change after the introduction of organic amendments to soil [96,97]. This suggests that changes occurred in the population of microbes capable of exploiting carbon substrates and also in this capability.

## 5. Conclusions

The application of both organic amendments in a greenhouse experiment induced an improvement in coriander growth and decreases in soil Cd and Zn bioavailability and plant Cd uptake through changes in soil properties, including OM enrichment. Despite a nearly 50% decrease in the Cd BCF values in the presence of organic amendments, the Cd accumulator phenotype of coriander was, regrettably, maintained. A synergistic effect of AMF inoculation and both organic amendments on decreasing extractable soil Cd and Zn was observed, unfortunately without reducing Cd accumulation in the shoots. Interestingly, vegetation and organic amendments improved the soil microbial biomass, enzyme activities, and metabolic potential, suggesting an improvement in soil quality and health. Our results indicate that organic amendments, contributing to the circular economy through waste recycling, and the biological amendment (AMF inoculation) can potentially provide long-term ecological benefits to TE-polluted soil and should be considered a reliable tool for future TE-polluted soil remediation. Further studies are needed to optimize the observed effects and to determine the mechanisms involved in the aided TE phytostabilization. In addition, aware that this pot experimentation is not necessarily a representative picture of plant’s performance and potential in field, future research should now be directed at the application of these amendments at field-scale levels.

## Figures and Tables

**Table 1:** Physicochemical properties of the initial soils after the amendment introduction and physicochemical properties of the amendments alone.

Parameters	Amended Soil Before Cultivation			Amendments		
	Non-Amended	Compost	Sewage Sludge	Compost	Sewage Sludge	Mycorrhizal Inoculum
**pH-H_2_O**	7.9 ± 0.00 a	7.9 ± 0.10 a	7.7 ± 0.05 a	7.11	8.92	8.3
**Clay (%)**	22.8 ± 0.71 a	22.45 ± 0.07 a	23.45 ± 0.35 ab	ND	ND	ND
**Silt (%)**	56.95 ± 0.21 a	56.45 ± 0.49 a	56.5 ± 0.14 a	ND	ND	ND
**Sand (%)**	20.2 ± 0.42 a	21.1 ± 0.28 a	20.1 ± 0.28 a	ND	ND	ND
**Total organic carbon (g/Kg)**	11.75 ± 1.20 a	16.1 ± 1.27 b	13.3 ± 0.85 ab	206.8	257.5	13.8
**Organic matter (g/Kg)**	19.4 ± 0.99 a	27.75 ± 2.19 b	22.85 ± 1.48 ab	355.7	442.8	23.8
**C/N**	10 ± 1.41 a	10 ± 0.01 a	7.5 ± 0.71 a	13	6	25
**CEC Metson (me/Kg)**	238.5 ± 4.95 a	248.5 ± 17.68 a	234.5 ± 4.95 a	328	167	524
**N (g/Kg)**	1.2 ± 0.14 a	1.55 ± 0.07 a	1.6 ± 0.14 a	15.1	40.9	0.5
**Available P Olsen (g/Kg)**	0.05 ± 0.01 a	0.09 ± 0.01 c	0.202 ± 0.02 b	0.91	2.85	0.03
**Available K (g/Kg)—K_2_O**	0.76 ± 0.01 a	0.72 ± 0.04 a	0.74 ± 0.00 a	1.55	0.78	1.66
**Available Mg (g/Kg)—MgO**	6.78 ± 0.13 a	7.86 ± 0.31 c	9.62 ± 0.65 b	1.66	1.75	1.05
**Available Ca (g/Kg)—CaO**	0.38 ± 0.04 a	0.43 ± 0.05 a	0.43 ± 0.01 a	12.84	25.5	10.72
**Cd (mg/Kg)**	5.29 ± 0.18	ND	ND	0.0	0.81	ND
**Cr (mg/Kg)**	32.18 ± 8.07	ND	ND	30.8	25.8	ND
**Cu (mg/Kg)**	21.29 ± 0.86	ND	ND	51.5	122	ND
**Ni (mg/Kg)**	24.46 ± 0.79	ND	ND	15.9	16.8	ND
**Pb (mg/Kg)**	326,84 ± 10.12	ND	ND	77.5	38.1	ND
**Zn (mg/Kg)**	409.80 ± 12.54	ND	ND	163	418	ND
**Germination index (%)**				63	58	ND

ND: not determined. Different letters indicate significant differences between the conditions (*p* < 0.05).

**Table 2:** Effects of organic amendment application and AMF inoculation on shoot dry weight (n = 30) and mycorrhizal rates (n = 3) of Coriander 3 months after planting. Different letters indicate significant differences between the treatments (*p* < 0.05).

		Biomass (mg/plant)	Mycorrhizal Rate (%)
Non-amended	NI	0.44 ± 0.24 a	53.6 ± 8.1 a
	I	0.47 ± 0.15 a	56.7 ± 3.5 a
Compost	NI	0.97 ± 0.26 c	54.54 ± 7.4 a
	I	0.65 ± 0.13 b	49.8 ± 8.0 a
Sewage sludge	NI	0.88 ± 0.40 bc	59.9 ± 1.3 a
	I	0.66 ± 0.41 ab	55.2 ± 9.3 a

**Table 3:** Biomass (µg/g dry soil) of specific microbial groups (Gram-positive bacteria, Gram-negative bacteria, saprotrophic fungi, and AMF), total microbial biomass, and stress indicator in the different soil treatments (n = 3).

			Gram-Positive Bacteria PLFA	Gram-Negative Bacteria PLFA	Fungal PLFA C18:2ω 6,9	AMF PLFA C16:1ω 5	Total Microbial ∑ PLFA	Stress Indicator
Non-amended	Non-vegetated	NI	1.44 ± 0.22 c	2.45 ± 0.39 c	0.80 ± 0.06 bc	0.81 ± 0.36 bc	5.85 ± 0.78 cd	0.69 ± 0.02 a
		I	1.43 ± 0.24 c	1.80 ± 0.14 c	0.84 ± 0.24 bc	0.56 ± 0.03 c	5.78 ± 0.43 d	0.38 ± 0.27 abc
	Vegetated	NI	1.83 ± 0.36 bc	2.87 ± 0.67 bc	0.82 ± 0.13 bc	0.97 ± 0.18 abc	7.28 ± 10.3 cd	0.52 ± 0.10 bcd
		I	2.29 ± 0.52 bc	3.40 ± 0.34 ab	1.36 ± 0.26 abc	0.96 ± 0.18 ab	8.17 ± 1.65 ab	0.28 ± 0.02 d
Compost	Non-vegetated	NI	1.50 ± 0.37 c	2.83 ± 1.41 bc	0.95 ± 0.19 abc	0.98 ± 0.20 abc	7.31 ± 1.46 bcd	0.58 ± 0.11 ab
		I	1.98 ± 0.44 bc	2.13 ± 0.24 c	0.65 ± 0.05 c	1.12 ± 0.35 ab	7.51 ± 0.88 cd	0.27 ± 0.08 abc
	Vegetated	NI	1.99 ± 0.09 abc	3.49 ± 0.32 ab	1.24 ± 0.45 abc	1.40 ± 0.44 a	8.13 ± 2.11 ab	0.50 ± 0.10 d
		I	2.21 ± 0.55 abc	3.50 ± 0.93 ab	1.32 ± 0.22 abc	1.08 ± 0.25 ab	8.67 ± 2.11 ab	0.26 ± 0.04 d
Sewage sludge	Non-vegetated	NI	2.01 ± 0.32 bc	2.74 ± 0.06 bc	0.86 ± 0.19 bc	0.73 ± 0.11 bc	6.79 ± 0.01 bcd	0.39 ± 0.02 bcd
		I	1.84 ± 0.96 ab	2.87 ± 1.14 bc	0.89 ± 0.47 ab	0.53 ± 0.22 bc	7.46 ± 1.43 bc	0.26 ± 0.02 ab
	Vegetated	NI	2.29 ± 0.63 bc	4.46 ± 0.65 a	1.61 ± 0.17 a	1.45 ± 0.43 a	10.28 ± 0.74 a	0.54 ± 029 d
		I	2.85 ± 1.14 a	4.05 ± 0.48 a	1.50 ± 0.28 ab	1.18 ± 0.11 ab	10.24 ± 1.78 a	0.31 ± 0.03 cd
Vegetation (V)			**	***	***	***	***	***
Mycorrhizal inoculation (M)			NS	NS	NS	NS	NS	NS
Amendments (A)			**	**	NS	NS	**	NS
M × V			NS	NS	NS	NS	NS	NS
M × A			NS	NS	NS	NS	NS	NS
V × A			NS	NS	NS	*	NS	NS
M × V × A			NS	NS	NS	NS	NS	NS

Different letters indicate significant differences between the treatments (*p* < 0.05). ***, **, and *: *p* < 0.001, 0.01, and 0.05, respectively. NS: not significant.

**Table 4:** Microbial functional diversity assessed by the total degradation activity (AWCD: average well color development), richness calculated from the substrate utilization pattern (31 substrates), and soil enzyme activities (μmol activity g soilDW/H) in the different soil treatments (n = 3).

			ACWD	Richness	Dehydrogenase	Arylsulfatase	Glucosidase	Cellubiosidase	Phosphomonoesterase
Non-amended	Non- vegetated	NI	19.24 ± 9.77 a	9.00 ± 2.73 a	5.69 ± 1.84 d	0.85 ± 0.03 b	1.95 ± 0.17 c	0.60 ± 0.09 b	2.37 ± 0.22 ab
		I	28.56 ± 8.79 a	10.11 ± 2.34 a	51.38 ± 14.61 d	0.68 ± 0.08 b	1.57 ± 0.52 c	0.52 ± 0.01 b	1.68 ± 0.06 b
	Vegetated	NI	95.44 ± 20.44 b	20.78 ± 1.35 b	131.08 ± 91.90 bcd	0.83 ± 0.04 b	1.64 ± 0.08 c	0.52 ± 0.00 ab	3.71 ± 1.51 ab
		I	91.90 ± 18.34 b	19.56 ± 3.56 b	216.09 ± 163.09 abcd	0.88 ± 0.10 ab	2.18 ± 0.31 bc	0.61 ± 0.03 ab	6.07 ± 0.06 a
Compost	Non- vegetated	NI	24.49 ± 9.64 a	9.22 ± 1.02 a	22.69 ± 11.49 d	0.85 ± 0.04 b	1.95 ± 0.27 bc	0.60 ± 0.01 ab	2.37 ± 0.38 ab
		I	24.23 ± 18.08 a	9.11 ± 6.55 a	52.44 ± 61.93 d	0.75 ± 0.06 b	2.10 ± 0.44 bc	0.55 ± 0.04 ab	2.78 ± 1.01 ab
	Vegetated	NI	110.42 ± 14.83 b	21.44 ± 1.90 b	343.61 ± 13.27 abc	0.86 ± 0.03 b	2.28 ± 0.35 bc	0.62 ± 0.02 b	3.37 ± 0.29 ab
		I	104.8 ± 17.19 b	20.89 ± 3.67 b	177.83 ± 95.44 bcd	0.83 ± 0.11 b	3.24 ± 1.34 ab	0.62 ± 0.06 bc	3.58 ± 0.73 ab
Sewage sludge	Non- vegetated	NI	104.8 ± 43.65 b	21.33 ± 2.73 b	76.88 ± 35.93 cd	0.86 ± 0.09 b	2.41 ± 0.26 bc	0.63 ± 0.05 ab	4.27 ± 0.72 ab
		I	109.84 ± 9.30 b	21.00 ± 0.58 b	107.70 ± 108.64 bcd	1.07 ± 0.08 a	3.29 ± 0.29 ab	0.70 ± 0.04 a	5.98 ± 0.96 a
	Vegetated	NI	136.88 ± 37.83 b	24.67 ± 3.79 b	355.29 ± 60.85 ab	0.77 ± 0.01 b	3.71 ± 1.21 a	0.62 ± 0.08 ab	5.13 ± 1.54 ab
		I	141.50 ± 5.66 b	24.67 ± 1.45 b	459.43 ± 204.7 a	0.79 ± 0.05 b	3.86 ± 1.54 a	0.62 ± 0.06	4.38 ± 1.28 ab
Vegetation (V)			***	***	***	NS	NS	NS	NS
Mycorrhizal inoculation (M)			NS	NS	NS	NS	NS	NS	NS
Amendments (A)			***	***	**	NS	**	**	*
M × V			NS	NS	NS	NS	NS	NS	NS
M × A			NS	NS	NS	**	NS	NS	NS
V × A			**	*	NS	**	NS	NS	NS
M × V × A			NS	NS	NS	**	NS	**	**

Different letters indicate significant differences between the treatments (*p* < 0.05). ***, **, and *: *p* < 0.001, 0.01, and 0.05, respectively. NS: not significant.

## Data Availability

All the data are included in the article and the Supplementary Materials.

## Supplementary Materials

The following supporting information can be downloaded at: https://www.mdpi.com/article/10.3390/microorganisms10112287/s1, Figure S1: Water pH in soil vegetated or not with coriander cultivated in the presence (I) or in the absence of AMF inoculum (NI) and/or without organic amendments (non-amended) or with compost or sewage sludge; Table S1: Total concentration of TE in soil vegetated or not (non-vegetated) with coriander cultivated in the presence or not of AMF inoculum (NI/I) and/or without organic amendments (non-amended) or with compost or sewage sludge; Table S2: Concentration of TE in shoots of coriander (µg g^−1^ dry matter (DM)) inoculated (I) or non-inoculated (NI) and cultivated in non-amended or amended (compost or sewage sludge) conditions.
