# Supplementary material for: Coriander (Coriandrum sativum L.) in Combination with Organic Amendments and Arbuscular Mycorrhizal Inoculation: An Efficient Option for the Phytomanagement of Trace Elements-Polluted Soils
Source: Microorganisms. 2022 Nov 17;10(11):2287. doi: 10.3390/microorganisms10112287 (PMC9692498; doi:10.3390/microorganisms10112287)
Supplement: Supplementary file 1 [file microorganisms-10-02287-s001.zip › microorganisms-2000081-supplementary.pdf]

Figure S1: Water pH in soil vegetated or not with coriander cultivated in presence (I) or in absence of AMF inoculum (NI) and/or without organic amendments (non-amended) or with compost or sewage sludges. Mean ± SD. Significant differences between treatments are indicated by different letters at the level of α=0.05.

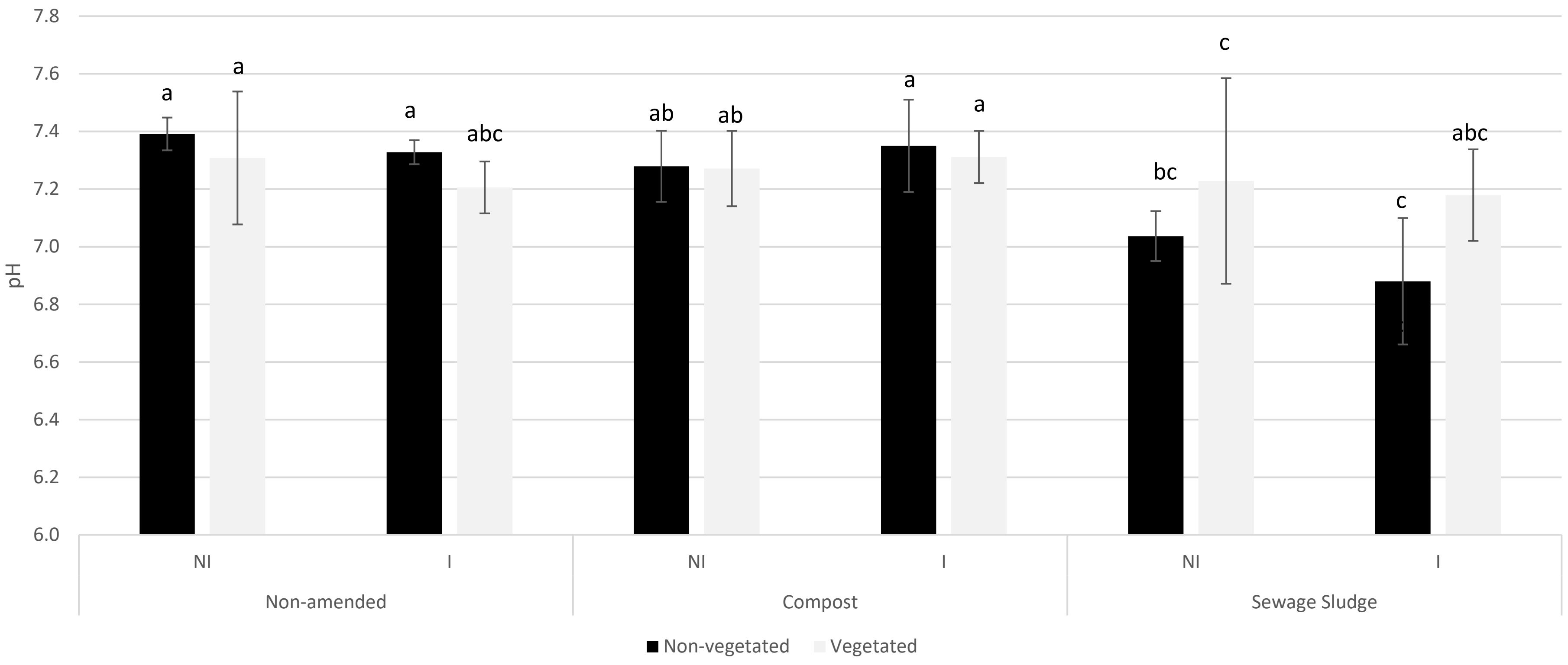

Table S1: Total TE concentrations (µg/g DS) in soil vegetated or not (non-vegetated) with coriander cultivated in the presence or not of AMF inoculum (NI/I) and/or without organic amendments (non-amended) or with compost or sewage sludge. Mean ± SD. Significant differences between treatments are indicated by different letters at the level of α=0.05.

			As	Cr	Cd	Cu	Ni	Pb	Zn
Non-amended	Non-vegetated	NI	<5	32.85 ± 0.57 a	4.92 ± 0.10 a	22.15 ± 0.29 a	28.92 ± 7.89 a	326.45± 13.70 a	405.94± 5.12 a
		I	<5	32.45 ± 0.56 a	4.83 ± 0.23 a	22.19 ± 0.83 a	22.46 ± 1.23 a	299.26± 10.42 ab	403.83± 17.96 a
	Vegetated	NI	<5	34.88 ± 1.96 a	4.90 ± 0.05 a	22.22 ± 0.31 a	20.51 ± 0.12 a	307.32± 2.50 bc	407.97± 2.61 a
		I	<5	31.85 ± 1.81 a	4.93 ± 0.23 a	22.91 ± 0.27 a	22.09 ± 0.76 a	304.19± 7.93 ab	411.5 1± 9.29 a
Compost	Non-vegetated	NI	8.9 ± 6.81	31.16 ± 1.72 a	5.04± 0.43 a	23.86 ± 3.45 a	29.58 ± 12.63a	301.05± 5.62 bc	404.65± 14.59 a
		I	<5	30.68 ± 2.23 a	5.00 ± 0.11 a	22.93 ± 1.64 a	19.55 ± 1.00 a	286.02± 9.33 bc	389.17± 16.53 a
	Vegetated	NI	8.20 ± 5.54	31.40 ± 2.28 a	4.68 ± 0.13 a	22.78 ± 0.65 a	21.55 ± 0.51 a	295.60± 5.77 bc	398.43± 12.58 a
		I	<5	33.79 ± 0.24 a	4.92 ± 0.34 a	24.19 ± 0.49 a	20.94 ± 0.38 a	300.77± 8.00 bc	405.70± 7.23 a
Sewage Sludge	Non-vegetated	NI	<5	36.63 ± 6.88 a	4.69 ± 0.15 a	24.66 ± 1.17 a	22.53 ± 4.20 a	280.20± 8.34 a	387.31± 12.45 a
		I	<5	31.48 ± 1.70 a	4.60 ± 0.12 a	25.05 ± 0.36 a	20.31 ± 0.23 a	296.52± 2.04 bc	397.87± 3.86 a
	Vegetated	NI	<5	34.35 ± 2.09 a	4.66 ± 0.04 a	24.80 ± 0.46 a	21.93 ± 0.61 a	296.01± 6.59 bc	399.73± 11.38 a
		I	<5	32.18 ± 2.33 a	4.82 ± 0.16 a	25.3 ± 1.54 a	20.69 ± 0.61 a	300.76± 6.88 bc	403.60± 7.87 a

Table S2: TE Concentrations (µg/g DM) in coriander shoots, inoculated (I) or non-inoculated (NI) cultivated in non-amended or amended (compost or sewage sludge) conditions. Mean ± SD. Significant differences between conditions are indicated by different letters at the level of α=0.05.

		As	Cr	Cd	Cu	Ni	Pb	Zn
Non-amended	NI	6.14 ± 0.25 a	1.23 ± 0.05 a	12.44 ± 4.54 a	5.91 ± 0.82 bc	1.38 ± 0.18 ab	7.39 ± 0.71 a	43.48 ± 13.95 a
	I	5.98 ± 0.19 a	1.20 ± 0.04 a	8.47 ± 0.52 ab	4.91 ± 1.12 bc	1.20 ±0.04 b	3.83 ± 0.95 ab	47.64 ± 6.31 a
Compost	NI	6.25 ± 0.16 a	1.25 ± 0.03 a	6.91 ± 1.16 ab	5.14 ± 0.49 bc	1.39 ± 0.28 ab	3.43 ± 0.76 b	45.35 ± 15.19 a
	I	6.23 ± 0.05 a	1.25 ± 0.01 a	6.38 ± 1.62 b	4.52 ± 0.87 c	1.63 ± 0.67 ab	2.92 ± 0.68 b	33.82 ± 5.15 a
Sewage sludge	NI	6.11 ± 0.28 a	1.22 ± 0.06 a	5.87 ± 0.84 b	10.18 ± 3.73 ab	1.24 ± 0.03 ab	4.04 ± 1.55 b	72.78 ± 27.39 a
	I	6.11 ± 0.26 a	1.22 ± 0.05 a	6.16 ± 1.71 b	11.86 ± 2.30 a	1.99 ± 0.74 a	3.25 ± 1.55 b	68.65 ± 13.72 a
